# Short-term lumbar disc and lumbar stability changes of one-hole split endoscope technique treatment of spinal stenosis

**DOI:** 10.1186/s12891-024-07443-9

**Published:** 2024-04-24

**Authors:** Jinghe Zhang, Ruqi Yan, Shidong Xu, Bin Shao, Yongfeng Dou

**Affiliations:** 1 Department of Spine Surgery, Binzhou Medical University Hospital, No. 661, Huanghe Er Road, Binzhou, Shandong 256603 China; 2 Department of Spine Surgery, Central Hospital of Zibo, No.54, Communist Youth League West Road, Zibo, Shandong 255020 China

**Keywords:** One-hole split endoscope technique, Lumbar spinal stenosis, Lumbar spinal decompression surgery, Biomechanics

## Abstract

**Objective:**

Investigating the early biomechanical effects of the one-hole split endoscope (OSE) technique on lumbar spine after decompression surgery.

**Methods:**

A retrospective analysis was conducted on 66 patients with lumbar spinal stenosis (LSS) who underwent OSE technique surgery at the affiliated hospital of Binzhou Medical University from September 2021 to September 2022. The patients had complete postoperative follow-up records. The mean age was (51.73 ± 12.42) years, including 33 males and 33 females. The preoperative and postoperative imaging data were analyzed, including disc height (DH), foraminal height (FH), lumbar lordosis angle (LLA), changes in disc angle, anterior-posterior translation distance, and lumbar intervertebral disc Pfirrmann grading. The visual analogue scale (VAS) was applied to evaluate the severity of preoperative, postoperative day 1, postoperative 3 months, and final follow-up for back and leg pain. The Oswestry Disability Index (ODI) was applied to assess the functionality at all the listed time points. The modified MacNab criteria were applied to evaluate the clinical efficacy at the final follow-up.

**Results:**

In 66 patients, there were statistically significant differences (*p* < 0.05) in DH and FH at the affected segments compared to preoperative values, whereas no significant differences (*p* > 0.05) were found in DH and FH at the adjacent upper segments compared to preoperative values. There was no statistically significant difference in the LLA compared to preoperative values (*p* > 0.05). Both the affected segments and adjacent upper segments showed statistically significant differences in Pfirrmann grading compared to preoperative values (*p* < 0.05). There were no statistically significant differences in the changes in disc angle or anterior-posterior translation distance in the affected or adjacent segments compared to preoperative values (*p* > 0.05). The VAS scores for back and leg pain, as well as the ODI, significantly improved at all postoperative time points compared to preoperative values. Among the comparisons at different time points, the differences were statistically significant (*p* < 0.05). The clinical efficacy was evaluated at the final follow-up using the modified MacNab criteria, with 51 cases rated as excellent, 8 cases as good, and 7 cases as fair, resulting in an excellent-good rate of 89.39%.

**Conclusions:**

The OSE technique, as a surgical option for decompression in the treatment of LSS, has no significant impact on lumbar spine stability in the early postoperative period. However, it does have some effects on the lumbar intervertebral discs, which may lead to a certain degree of degeneration.

## Introduction

Lumbar spinal stenosis (LSS) is a common degenerative disease in spine surgery, characterized by symptoms such as lower limb pain and intermittent claudication due to nerve compression. For cases where conservative treatment is ineffective or symptoms progressively become worse, surgical intervention is frequently required [1]. Compared to traditional open decompression surgery, which has drawbacks such as significant damage to the lumbar posterior column and a major impact on lumbar biomechanics [2], endoscopic treatment of lumbar spinal stenosis offers advantages such as minimal invasiveness and a shorter surgical time [3]. Consequently, it has become a preferred approach for treating LSS. In recent years, emerging techniques like the one-hole split endoscope (OSE) have shown advantage. Compared to well-established endoscopic techniques such as percutaneous endoscopic lumbar discectomy [4] and unilateral biportal endoscopic discectomy [5], OSE offers similar advantages, including minimal surgical trauma, reduced blood loss, and more rapid postoperative recovery [6]. Additionally, OSE provides greater flexibility and eliminates errors and blind spots associated with limited viewing angles. Zhang et al. [7] showed that the hospitalization time, operative duration, intraoperative blood loss, number of fluoroscopy exposures of OSE technique in single-stage spinal canal decompression were not different from those of other endoscopic methods, but the incision length was smaller and the damage to muscle tissue was less.

Although the OSE technique offers many advantages, it inevitably requires the removal of a portion of the facet joint and joint capsule structures during spinal canal decompression. The facet joints, together with the lumbar intervertebral disc, contribute to the complex motion of the lumbar spine in three dimensions and six directions, playing a certain role in lumbar stability [8]. A biomechanical study demonstrated that damage to the lumbar posterior column can lead to changes in stress distribution in the responsible and adjacent intervertebral discs [9]. It is evident that injury to the facet joints can impact the lumbar stability and intervertebral disc health. Currently, there are few clinical studies reporting on the effects of the OSE technique on lumbar biomechanics following spinal canal decompression. Therefore, investigating the early postoperative changes in lumbar biomechanics resulting from OSE surgery for LSS and their impact on lumbar stability and disc degeneration holds certain clinical significance. In this study, we retrospectively analyzed a total of 66 cases of lumbar spinal stenosis treated by the OSE technique in the Department of Spine Surgery at Binzhou Medical University Affiliated Hospital, with complete postoperative follow-up records. A comparison was made between the preoperative and early postoperative changes in lumbar biomechanics.

## Materials and methods

This study was approved by the Ethics Committee of Binzhou Medical University, with approval number KYLL-2022-159.

### Inclusion and exclusion criteria

Inclusion criteria: (1) Magnetic resonance imaging (MRI) and computerized tomography (CT) examinations indicating single-segment spinal stenosis with Schizas grade [10] ≥ B. (2) Clinical symptoms and signs consistent with imaging findings. (3) History of intermittent claudication and presence of neurogenic symptoms such as leg pain and numbness. (4) Inadequate response to 6 months of conservative treatment and severe impact on quality of life.

Exclusion criteria: (1) Patients with Meyerding grade I or above, lumbar spondylolisthesis, or lumbar spinal instability (LSI) (diagnosed by measuring the angle change and translation distance on flexion-extension X-rays, with angle change > 15° and translation distance > 3 mm as criteria for LSI [11]). (2) Patients with scoliosis. (3) Patients with a history of previous lumbar spine surgery. (4) Patients with coexisting other lumbar spine-related diseases, such as spinal canal infection, spinal tumor, or tuberculosis, among other. (5) Patients with symptoms that cannot tolerate surgical treatment. (6) Patients with comorbid psychiatric disorders or other conditions that hinder treatment and assessment.

### General information

A total of 66 eligible patients were included, with a mean age of (51.73 ± 12.42) years. Among them were 33 males and 33 females. Among the patients, 25 had lumbar disc herniation. The surgical segments were as follows: L3/4 in 9 cases, L4/5 in 30 cases, and L5/S1 in 27 cases. Among them, 51 patients underwent unilateral decompression, while 15 patients underwent one-hole split endoscope-unilateral laminotomy biportal decompression bilateral decompression. There were 15 cases with coexisting hypertension and 8 cases with diabetes, both of which were well controlled(Table 1).

**Table 1:** General information of the patients

General information	Surgical Statistics(n = 66)	
Age	51.73 ± 12.42	
Gender		
Male	33	
Female	33	
Operative level		
L3/4	9	
L4/5	30	
L5/S1	27	
Decompression method		
Unilateral decompression	51	
Bilateral decompression	15	
Comorbidities		
Hypertension	15	
Diabetes	8	
Perioperative data		
Operative time(min)	66.29 ± 8.68	

### Surgical technique

**Fig. 1:**
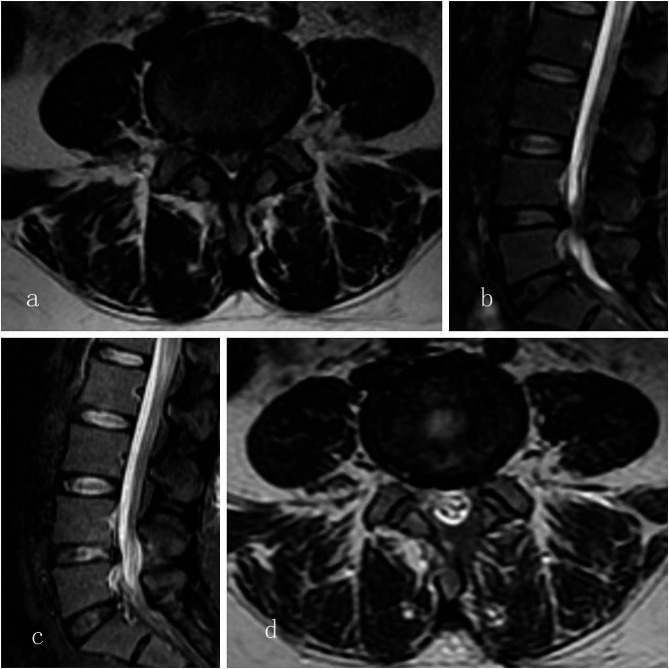
**a** and **b** show preoperative horizontal and sagittal MRI images, respectively, while c and d represent postoperative horizontal and sagittal MRI images, respectively, indicating a significant increase in the cross-sectional area of the spinal canal

**Fig. 2:**
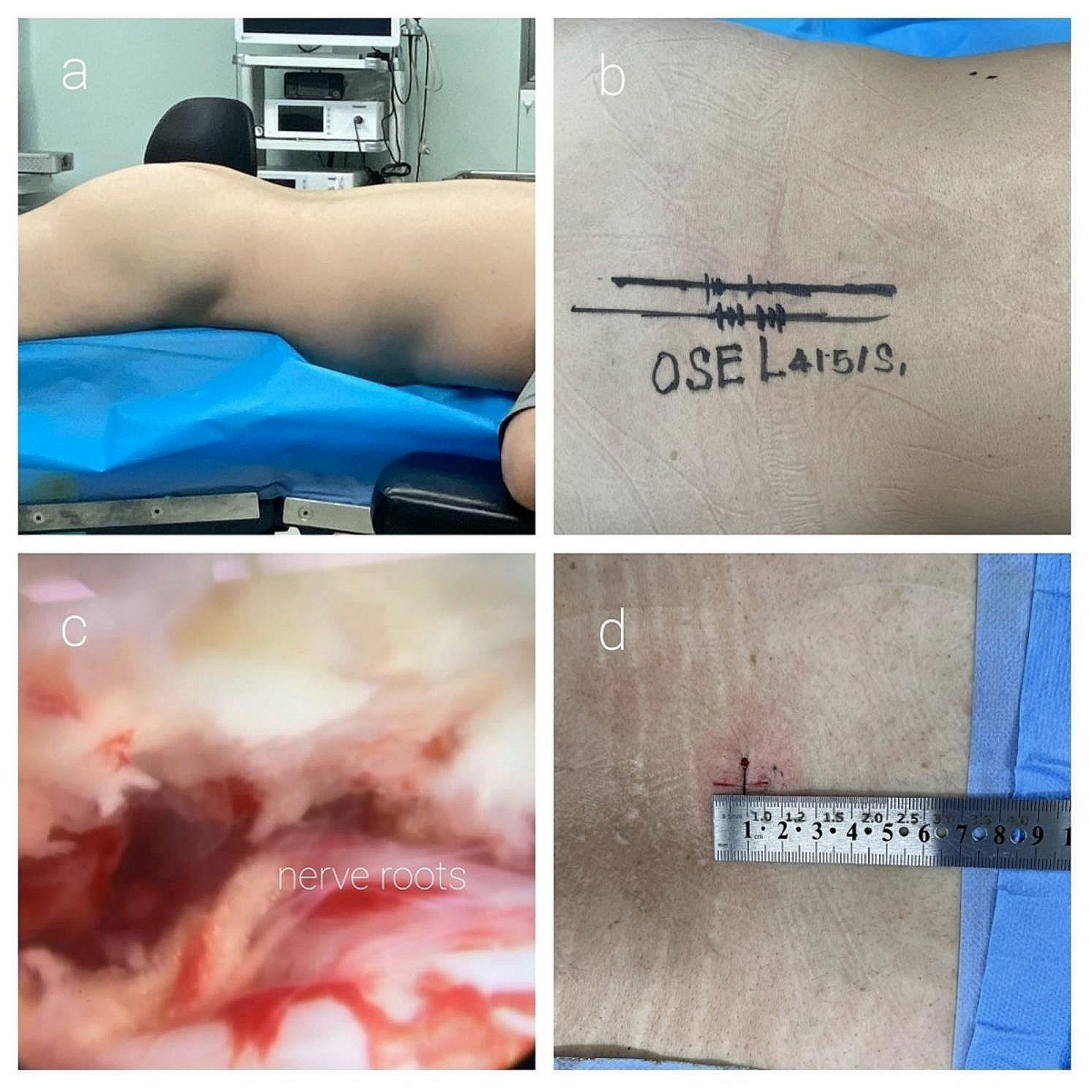
**a**: The body placement; **b**: The surgical marking; **c**: The endoscopic fields during operation; **d**: The postoperative incision

The surgeries were performed by the same surgeon DYF. All patients underwent general anesthesia with endotracheal intubation and inhalation anesthesia. Patients were positioned prone on the operating table with the operative area maintained in a horizontal position and the abdomen suspended. Under fluoroscopic guidance, Kirschner wires were used to locate the target intervertebral space. A longitudinal incision of approximately 10 mm was made 15 mm to the posterior midline, followed by sequential dilators to create a working channel. Endoscopes and plasma radiofrequency electrodes were inserted through the working channel. Hemostasis was achieved using plasma radiofrequency coagulation. Soft tissues within the intervertebral space were cleared using a burr and bone rongeurs, removing part of the lamina, spinous process base, and ligamentum flavum. Partially fused articular processes were resected. The dural sac and nerve roots were exposed, and the nerve root mobility was assessed and found to be satisfactory. After thorough hemostasis and irrigation using a plasma radiofrequency ablation tip, no active bleeding was observed in the field. The incision was sutured, and the surgery was completed. (Figures 1 and 2)

### Postoperative treatment

After surgery, patients were advised to wear a lumbar brace for 48 h and perform mobilization activities while avoiding excessive weight-bearing and excessive movement of the lumbar spine. The lumbar brace should be worn for 4–6 weeks after discharge from the hospital. Within 3 months after surgery, patients should avoid vigorous activities and excessive weight-bearing. Regular follow-up examinations, including lumbar spine X-rays (anteroposterior and lateral views), dynamic X-rays, lumbar spine CT scan, and lumbar spine MRI, should be performed at regular intervals up to 1 year post-surgery.

### Evaluation criteria

Pain assessment using the Visual Analogue Scale (VAS) [12] was conducted to evaluate the preoperative, postoperative (1 day), 3-month postoperative, and final follow-up assessments of lumbar and leg pain, with the results recorded. Functional assessment using the Oswestry Disability Index (ODI) [13] was conducted to evaluate the preoperative, 3-month postoperative, and final follow-up assessments of functionality, with the results recorded. The modified MacNab criteria was applied for the final follow-up evaluation of surgical efficacy.

**Fig. 3:**
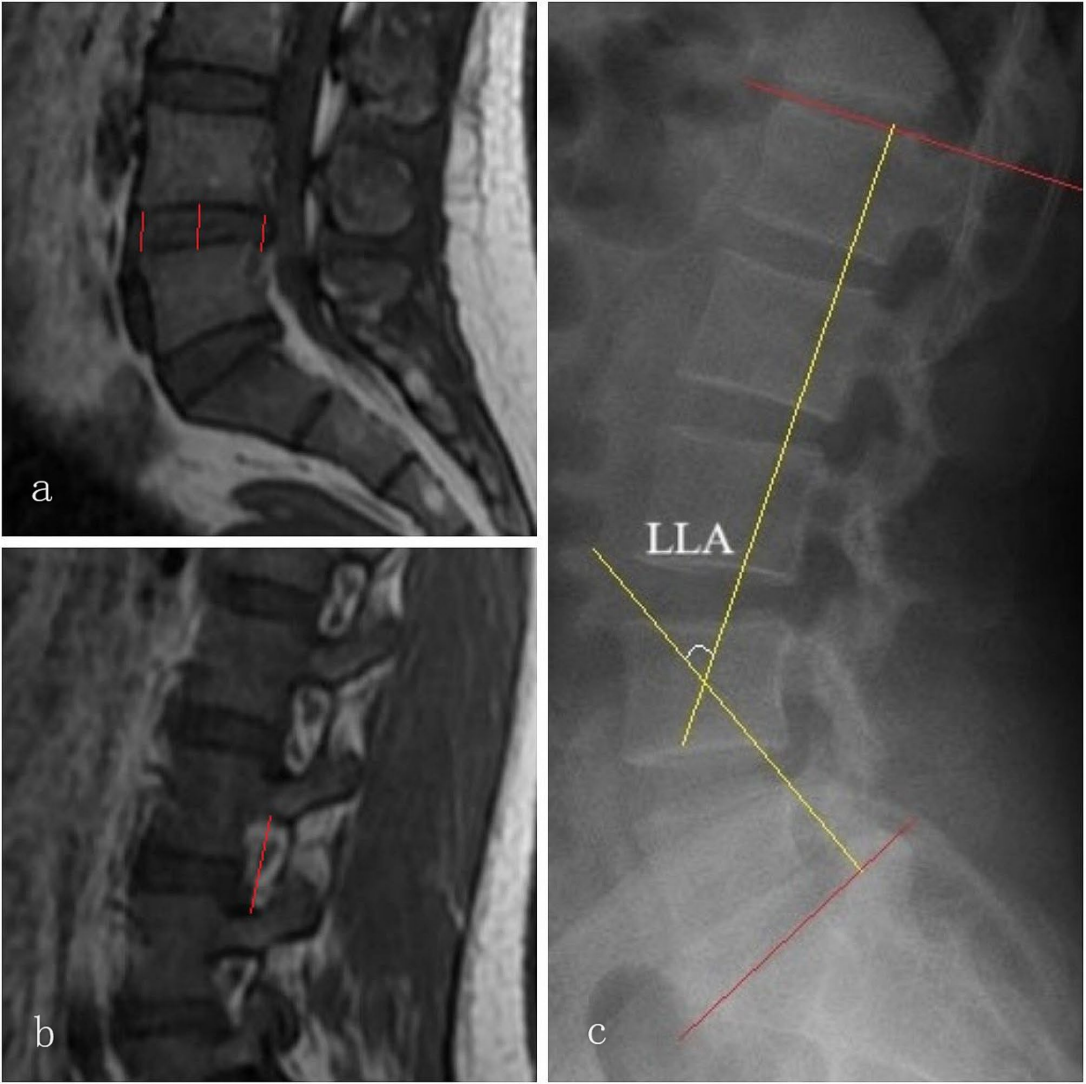
**a**, **b**, and **c** represent measurements of DH, FH, and the LLA, respectively

**Fig. 4:**
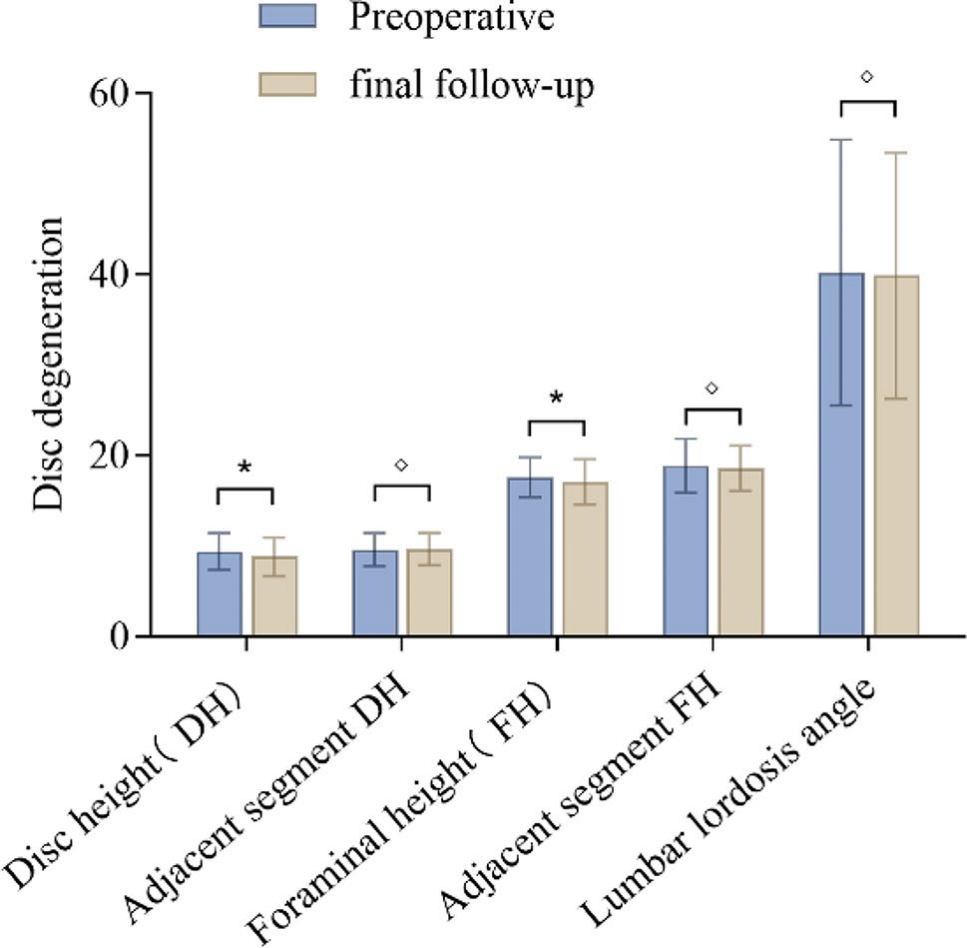
Data on intervertebral disc degeneration in each group. “*” indicates a significant difference in data (*p* < 0.05), while “◇” indicates no significant difference in data (*p* > 0.05)

Imaging evaluation: Measurements of preoperative and 1-year postoperative imaging data were conducted by YRQ and another radiologists from Binzhou Medical University Affiliated Hospital. The measured segments included the responsible segment and the adjacent proximal segment, and the two radiologists were blind to the purpose and methods of this study. The measurement indicators included: (1) Disc height (DH): measurement of the anterior, central, and posterior heights of the corresponding intervertebral disc on the mid-sagittal plane of the lumbar spine MRI, with the average value measured as the DH [14]; (2) Foraminal height (FH): measurement of the maximum distance between the upper and lower edges of the bilateral vertebral pedicles, and the average value measured as the FH [14]; (3) Lumbar lordosis angle (LLA): was determined using the Cobb method, by measuring the angle between the upper endplate of L1 and the sacral endplate (S1), which represents the LLA [15] (Fig. 3); (4) X-ray evaluation of lumbar hyperextension or hyperflexion positions and measurement of the difference in the intervertebral disc angle and the anterior-posterior translation distance [11]; (5) Pfirrmann grading of the responsible segment and the adjacent proximal segment intervertebral discs were statistically analyzed and recorded [16].

### Statistical analysis

The data were processed using SPSS 25.0 statistical software. Descriptive statistics, such as mean ± standard deviation, were used to represent continuous variables. For the comparison of data before and after surgery, the differences were calculated and tested for normality using the Shapiro-Wilk test. If the differences were normally distributed, a paired t-test was applied. If the differences did not follow a normal distribution, the Wilcoxon signed-rank test was used. For the comparison of data at multiple time points, a one-way repeated measures analysis of variance was applied, followed by post-hoc multiple comparisons after surgery. If the assumption of sphericity, tested using Mauchly’s test of sphericity, was violated, the Greenhouse-Geisser correction method was applied. Bonferroni correction was used for pairwise comparisons between different time points. Categorical data were analyzed using the chi-square test. A p-value less than 0.05 was considered to be statistically significant for all analyses.

## Results

### Clinical and functional outcomes

All 66 patients underwent surgery successfully without any need for subsequent surgeries and completed the planned postoperative follow-up of up to 1 year post surgery.

At each postoperative time point, both the VAS scores and ODI significantly improved compared to the preoperative values (Table 2). The pairwise comparisons at different time points were statistically significant (*p* < 0.05), (Tables 3, 4 and 5). The surgical outcomes were evaluated using the modified MacNab criteria at the final follow-up. Out of the 66 patients, 51 had an excellent outcome, 8 a good outcome, and 7 a fair outcome, resulting in an overall success rate of 89.61%.

**Table 2:** Comparison of the VAS scores and ODI at different time points before and after surgery

Index	Preoperative	Postoperative 1 day	Postoperative 3 months	Last follow-up	Statistic Value	P-value
Low back pain VAS score (points)	7.00 ± 0.74	2.98 ± 0.73	2.20 ± 0.87	1.76 ± 0.70	F=23156.00	<0.001
Leg pain VAS score (points)	6.85 ± 0.83	2.77 ± 0.74	2.03 ± 0.96	1.62 ± 0.67	F=2129.61	<0.001
ODI (%)	62.73 ± 7.46	——	26.57 ± 6.61	15.25 ± 2.58	F = 1187.84	<0.001

**Table 3:** Pairwise comparison of the leg pain VAS scores at different time points

Index		95% CI of the difference	P-value
Preoperative	Postoperative 1 day	3.917–4.235	<0.001
	Postoperative 3 months	4.624–5.012	<0.001
	Last follow-up	4.994–5.461	<0.001
Postoperative 1 day	Postoperative 3 months	0.584–0.901	<0.001
	Last follow-up	0.946–1.357	<0.001
Postoperative 3 months	Last follow-up	0.167–0.651	<0.001

**Table 4:** Pairwise comparison of the low back pain VAS scores at different time points

Index		95% CI of the difference	P-value
Preoperative	Postoperative 1 day	3.974–4.056	<0.001
	Postoperative 3 months	4.656–4.950	<0.001
	Last follow-up	5.086–5.339	<0.001
Postoperative 1 day	Postoperative 3 months	0.650–0.926	<0.001
	Last follow-up	1.086–1.369	<0.001
Postoperative 3 months	Last follow-up	0.252–0.626	<0.001

**Table 5:** Pairwise comparison of the ODI (%) at different time points

Index		95% CI of the difference	P-value
Preoperative	Postoperative 3 months	0.329–0.394	<0.001
	Last follow-up	0.450–0.499	<0.001
Postoperative 3 months	Last follow-up	0.092–0.135	<0.001

### Imaging results

**Fig. 5:**
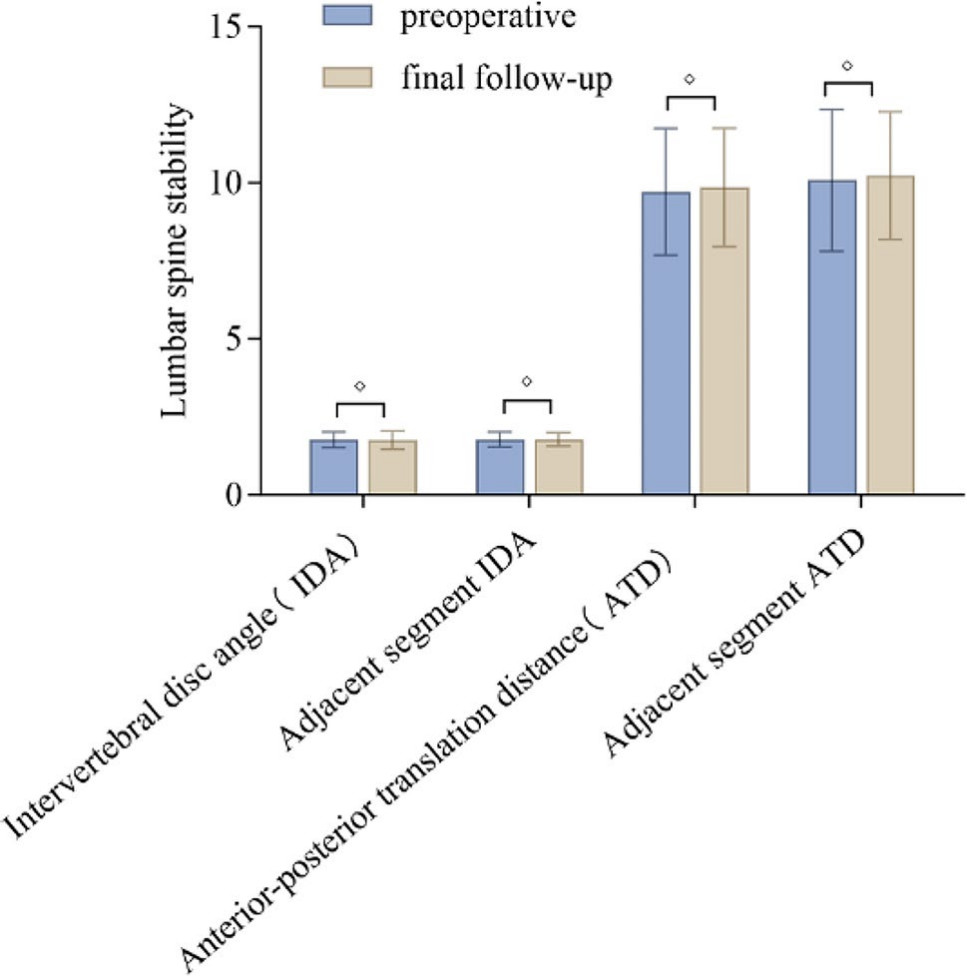
Data on lumbar spine stability in each group. “*” indicates a significant difference in data (*p* < 0.05), while “◇” indicates no significant difference in data (*p* > 0.05)

There was a statistically significant difference in the DH and FH of the affected segments compared to the preoperative values (*p* < 0.05). However, there was no statistically significant difference in the DH or FH of the adjacent segments compared to the preoperative values (*p* > 0.05), although there was a decrease in mean values postoperatively. The difference in the LLA compared to the preoperative values was not statistically significant (*p* > 0.05), (Fig. 4). There was a statistically significant difference in the Pfirrmann grading between the affected segments and the adjacent segments compared to the preoperative values (*p* < 0.05). The changes in the intervertebral space angle and anterior-posterior translation distance of the affected segments and adjacent segments in hyperextension and hyperflexion positions on lateral X-ray images compared to the preoperative values did not have a statistically significant difference (*p* > 0.05), (Table 6; Fig. 5).

**Table 6:** Comparison of radiological parameters before and after surgery

Index	Preoperative	Last follow-up	Statistic Value	P-value
DH($\bar{x} \pm s$, mm)	9.39 ± 2.04	8.81 ± 2.12	t=3.207	0.002
the adjacent upper segment DH($\bar{x} \pm s$, mm)	9.60 ± 1.81	9.65 ± 1.76	t=-0.371	0.712
FH($\bar{x} \pm s$, mm)	17.57 ± 2.19	17.07 ± 2.52	t=2.003	0.049
the adjacent upper segment FH($\bar{x} \pm s$, mm)	18.84 ± 2.98	18.58 ± 2.50	t=1.046	0.299
LLA($\bar{x} \pm s$, °)	40.19 ± 14.68	39.89 ± 13.59	t=0.322	0.749
Pfirrmann grading(example, 1/2/3/4/5)	26/24/6/5/5	15/23/13/9/6	χ^2^=6.785	0.148
the adjacent upper segment Pfirrmann grading(example, 1/2/3/4/5)	28/23/7/5/3	16/21/11/10/8	χ^2^=8.192	0.085
intervertebral disc angle($\bar{x} \pm s$, °)	9.71 ± 2.03	9.85 ± 1.89	t=-0.571	0.570
the adjacent upper segment intervertebral disc angle($\bar{x} \pm s$, °)	10.08 ± 2.27	10.23 ± 2.05	t=-0.559	0.578
anterior-posterior translation distance($\bar{x} \pm s$, mm)	1.78 ± 0.25	1.76 ± 0.29	t=0.697	0.488
the adjacent upper segment anterior-posterior translation distance($\bar{x} \pm s$, mm)	1.79 ± 0.25	1.79 ± 0.22	t=-0.155	0.877

## Discussion

OSE, as an emerging technique for treating LSS, has now gained recognition among most physicians [7]. However, the OSE technique still poses the issue of partial removal of lamina and facet joint articulations. Finite element analysis has shown that partial removal of facet joint articulations can increase lumbar spine mobility and stress on intervertebral discs [17]. Studies by Ruberté et al. [18] have indicated that facet joint degeneration and injury can significantly weaken load-bearing function, shifting the mechanical center of the lumbar complex forwards towards the intervertebral disc and increasing stress on the disc. Excessive compressive stress on the intervertebral disc tissues is an important factor in lumbar intervertebral disc degeneration [19], as supported by animal models established by Guo et al. [20]. Adams et al. [21] argued that excessive mechanical load disrupts the structure of the intervertebral disc, leading to irreversible cellular-mediated cascade reactions and disc degeneration. Therefore, it can be inferred that changes in intervertebral disc stress contribute to disc degeneration to a certain extent.

In systematic evaluations of intervertebral disc degeneration, Pfirrmann grading [16] has been recognized as an effective and reliable method for assessing disc degeneration by MRI [22]. The results of the present study showed significant differences in Pfirrmann grading between the affected lumbar intervertebral disc and the adjacent upper lumbar intervertebral disc before and after surgery, indicating a certain degree of disc degeneration. Teichtahl et al. [23] demonstrated a dose-response relationship between DH and disc degeneration, suggesting that a decrease in DH indicates the occurrence of disc degeneration. In the present study, there were significant differences in DH of the affected disc after surgery compared to before surgery (*p* < 0.05), while the differences in DH of the adjacent upper disc compared to before surgery were not statistically significant (*p* > 0.05), although the mean DH decreased in both cases. Moreover, FH is closely related to intervertebral disc and vertebral body height, while foramen width(FW)is related to the length of the spinal canal and pedicles [24]; narrowing of the intervertebral space can lead to a decrease in FH. Cinotti et al. [25] found through cadaveric studies that disc removal resulted in a decrease in FH but no apparent change in FW. FH is positively correlated with DH, with which with the results of the present study are consistent, where FH decreased significantly after surgery (*p* < 0.05). When FH decreases, the risk of nerve root compression in the narrowed neural foramen increases. By contrast, in the present study, the VAS scores and ODI improved compared to before surgery, and there were no cases of increased scores due to nerve root compression. In the sagittal plane, nerve roots are located in the upper part of the neural foramen, where FH is larger. Therefore, even with a minor decrease in FH, nerve root compression is unlikely to occur. It was observed that after OSE surgery for spinal decompression, there was an early reduction in DH and FH, leading to a certain degree of disc degeneration.

Spinal sagittal balance is an important postural characteristic, and its functional and clinical importance are increasingly recognized [26, 27]. Many studies have evaluated the correlation between the LLA and spinal degeneration [28, 29]. Most researchers believe that there is a significant correlation between the LLA and isthmic spondylolisthesis. However, a study [30] found no correlation between the LLA and disc herniation or lumbar degeneration, indicating a need for further research to investigate the relationship between the LLA and disc herniation or lumbar degeneration. This is consistent with the results of the present study, which found no statistically significant difference in the LLA after OSE surgery compared to before surgery in the 66 patients. This indicated that OSE surgery for lumbar spinal decompression does not cause changes in the LLA during the early postoperative period.

Lumbar segmental instability refers to abnormal movement of the lumbar spine under physiological loading, with a range of motion greater than the normal range [11, 31]. It has been found that removal of the facet joints can affect the stability of the corresponding lumbar segment [32]. The most commonly accepted criteria for diagnosing lumbar instability [11] include a change in intervertebral space angle > 15° and an anterior-posterior translation distance > 3 mm in flexion and extension X-rays of the lumbar spine. Previous studies have shown that lumbar pain is the main manifestation of lumbar instability, particularly during activity [33]. In the present study, there were no statistically significant differences in the difference in the intervertebral space angle or anterior-posterior translation distance between the affected segment and adjacent segments in flexion or extension X-rays before or after surgery (*p* > 0.05). The VAS scores for lumbar pain improved at all follow-up time points compared to before surgery, and there were no cases of worsened pain, indicating the absence of lumbar instability in the patients included in this study. As one of the surgical treatment options for lumbar spinal stenosis, the OSE technique can effectively preserve lumbar stability.

On the basis of the above analysis, the use of the OSE technique for decompression surgery in LSS has minimal early postoperative effects on lumbar stability, which is consistent with previous studies [7]. However, a finite element analysis conducted by Shi et al. [9] suggested that removal of the lumbar facet joints may cause changes in intervertebral disc stress. Additionally, the present study found that surgery had a certain impact on the intervertebral discs of the patients, which was consistent with the results of Sairyo et al. [34].

This study had a relatively short follow-up time and a small sample size. Further research and observation are needed to evaluate the long-term effects of the OSE technique on lumbar stability and its impact on disc degeneration. In this paper, the extent of resection was not grouped, and the relationship between the extent of resection and the degree of disc degeneration was not explored.

## Conclusion

As a surgical technique for treating LSS, the OSE technique provides satisfactory early clinical outcomes and has no significant impact on lumbar stability in the early postoperative period. However, it does have a certain impact on the intervertebral discs and may lead to a degree of disc degeneration.

## Data Availability

The datasets generated during and/or analysed during the current study are available from the corresponding author on reasonable request.

## Abbreviations

OSE: One-hole split endoscope
LSS: Lumbar spinal stenosis
MRI: Magnetic resonance imaging
CT: Computerized tomography
LSI: Lumbar spinal instability
VAS: Visual Analogue Scale
ODI: Oswestry Disability Index
DH: Disc height
FH: Foraminal height
LLA: Lumbar lordosis angle
IDA: Intervertebral disc angle
ATD: Anterior-posterior translation distance
FW: Foramen width
